# Potential Errors in CMAQ NO:NO_2_ Ratios and Upper Tropospheric NO_2_ Impacting the Interpretation of TROPOMI Retrievals

**DOI:** 10.1021/acsestair.4c00198

**Published:** 2025-04-29

**Authors:** Abiola S. Lawal, T. Nash Skipper, Cesunica E. Ivey, Daniel L. Goldberg, Jennifer Kaiser, Armistead G. Russell

**Affiliations:** 1 School of Civil & Environmental Engineering, 122528 Georgia Institute of Technology, 790 Atlantic Drive, Atlanta, Georgia 30332-0355, United States; 2 Department of Civil and Environmental Engineering, 760 Davis Hall, 172443 University of California, Berkeley, California 94720-1710, United States; 3 Department of Environmental and Occupational Health, George Washington University, 950 New Hampshire Avenue NW, Washington, D.C. 20052, United States; 4 School of Earth and Atmospheric Sciences, Georgia Institute of Technology, 790 Atlantic Drive, Atlanta, Georgia 30332-0355, United States

**Keywords:** satellite retrievals, TROPOMI, NO_2_ vertical column densities, NO_*x*_ ratio, power plants, chemical transport models

## Abstract

Although Chemical Transport Models (CTMs) such as the Community Multiscale Air Quality Model (CMAQ) have been used in linking observations of trace gases to emissions and developing vertical column distributions, there remain consistent biases between CTM simulations and satellite retrievals. Simulated tropospheric NO_2_ vertical column densities (VCDs) are generally higher over areas with large NO_*x*_ sources when compared with retrievals, while an opposite bias is found over low NO_*x*_ regions. Artificial (i.e., numerical) dilution in the model, where emissions are mathematically dispersed uniformly within the originating CTM grid, can impact modeled NO:NO_2_ ratios, while lower CTM VCD levels often observed over rural areas can be attributed to missing emission sources of NO_*x*_ or flawed horizontal/vertical transport. Potential causes of both low and high biases are assessed in this study using CMAQ and Tropospheric Monitoring Instrument (TROPOMI) NO_2_ retrievals. It was found that more detailed modeling of NO_*x*_ plumes to assess the NO:NO_2_ ratio in two power plant plumes can mitigate the effect of artificial computational dilution, reducing the bias and overall differences in the observed vs modeled plumes (errors reduced by 30%). Adjustments of upper tropospheric NO_2_ led to overall improvements, with a reduction in CMAQ bias (−43% to −29%) and improved spatial correlation (0.81 to 0.86). This study highlights the importance of having accurate modeled NO:NO_2_ ratios when comparing models to retrievals and the impact of unintentional numerical dilution.

## Graphical abstract

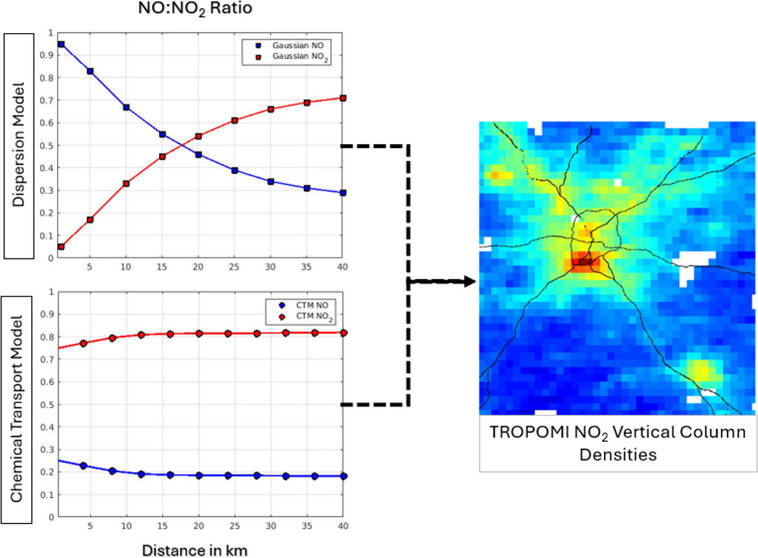

## 1 Introduction

Comparing satellite-derived nitrogen dioxide (NO_2_) fields with chemical transport model (CTM) results has proven to be a powerful approach in identifying potential errors in estimated nitrogen oxide (NO_*x*_ = NO + NO_2_) emission inventories.^1–4^ Before 2018, satellite-derived fields were relatively coarse (i.e., about 13 km or more). The new Tropospheric Monitoring Instrument (TROPOMI) has provided much finer resolution fields (approximately 4 km with oversampling on the order of 1 km)^5^ since then. These fields have been compared with similarly fine-scale regional CTMs such as the Community Multiscale Air Quality Model (CMAQ)^6^ to identify potential errors in emissions estimates. However, comparing modeled and TROPOMI NO_2_ vertical column densities (VCDs) to identify potential error sources in NO_*x*_ emissions assumes that the modeled NO to NO_2_ ratio is correct.^7^ If the ratio is not accurate, there will be a mismatch between observed and CTM NO_2_ VCDs, even if the total modeled NO_*x*_ in a column is accurate.

CTMs such as CMAQ are used when comparing modeled and observed VCDs because they can capture NO to NO_2_ conversion in the atmosphere and provide simulated vertical distributions of NO_2_ for calculating air mass factors (AMFs) used for diagnosing satellite retrievals.^8^ Studies have found that satellite observations and modeled VCDs show similar spatial distributions, supporting the use of satellite retrievals for assessing NO_*x*_ emissions inventories.^9^

However, in most of these high-resolution (i.e., ≤4 km) studies, CTM NO_2_ VCDs are higher than TROPOMI VCDs near large NO_*x*_ emission sources (e.g., roadways, urban centers, power plants, and airports) with differences as high as 50–60%.^10–13^ Some of these sources, such as power plants, have continuous emission monitoring systems (CEMs), common for electricity generating units (EGUs), such that the emissions from those sources are accurately known, and thus it may be assumed that errors in CTM NO_*x*_ concentrations would be significantly minimized. This would suggest that such sources with CEMs can be used to assess potential biases in satellite retrievals with minimal error;^14^ however, the high model bias relative to satellite retrievals for these sources still exists.

One issue that impacts the retrieval comparison with Eulerian models such as CMAQ^15^ and GEOS-Chem^16^ when no plume-in-grid treatment is incorporated^17^ is the immediate dispersion (“well mixed”) of point source emissions within the computational grid cell of the source. The immediate dilution of NO_*x*_ into the computational cell itself does not impact the total columnar abundance of NO_*x*_, but the overdilution allows the NO to react more rapidly with ozone (O_3_) to form NO_2_. This is because the artificial dilution increases the relative amount of ozone to NO_*x*_ available for reaction, leading to more NO being oxidized to NO_2_ in CTMs. This numerical artifact would be most severe over computational grid sizes that are much larger than the plume width (e.g., >1 km horizontal spacing) so that much more NO is converted to NO_2_ ^18^ in the model.

In the actual plume, plume NO_2_ concentrations are affected by chemical kinetics^19^ and thereby the relative initial concentrations of NO_*x*_ and O_3_. Here, the large abundance of NO (which makes up 90–95% of fresh NO_*x*_ emissions)^7^ will react to deplete the ozone in the plume such that near the source most of the plume NO_*x*_ will continue to be found as NO since there is no more available ozone to react within the core of the plume. In this case, O_3_ serves as the limiting reagent, hindering further conversion of NO_*x*_ to NO_2_.

The bias becomes larger when finer scale retrievals and model simulations are compared versus when using larger grids. This is because the modeled NO_*x*_ levels along the plume are higher when using finer grids.^20^ Thus, while the advent of having fine scale (e.g., <5 km) NO_2_ fields from TROPOMI has led to being able to conduct more detailed and direct comparisons between satellite observations and modeled results, the smaller control volume actually exacerbates the problem of having a higher modeled NO_2_:NO ratio in the plume than actually occurs.^18,21^ The well-mixed assumption near the source in smaller grids that have less diluted modeled NO_2_, in combination with artificial dilution of subgrid scale emissions over larger grids, results in higher modeled NO_2_ levels, with more NO_*x*_ being simulated as NO_2_ instead of NO. This compounds the errors associated with differences in model resolution that influence the nonlinear chemistry of NO_*x*_.^18,20^

A closely associated issue impacting the comparison of modeled vs observed NO_2_ VCDs is that most CTMs rapidly diffuse high pollutant concentrations horizontally and vertically^22,23^ such that the plumes are quickly diffused not only in the single computational cell where they are emitted but also throughout the entire column that is within the mixed layer, which can be up to 1000 m thick. This also extends to horizontally adjacent grids, as well. Thus, the plume is computationally highly diluted almost immediately, and there is sufficient ozone to convert most of the emitted NO to NO_2_, further increasing the NO_2_ VCD differences between CTMs and retrievals.

In this study, the potential contribution from artificial dilution (vertical and horizontal diffusion of the plume) in CMAQ and differences from tropospheric TROPOMI NO_2_ VCD are assessed using a Gaussian plume model linked to a NO–NO_2_–O_3_ pseudosteady state (PSS) model. The focus here is on plumes from high NO_*x*_, elevated point sources, primarily EGUs.^24^ In high NO_*x*_ plumes, the concentrations of oxidizing species such as O_3_ and OH will be reduced due to the high NO and low organic carbon levels. Also considered in this study is an adjustment to simulated NO_2_ in the upper troposphere to account for low model biases found in the upper troposphere, potentially due to a bias in the tropospheric/stratospheric exchange, underestimates of emission sources (i.e., aircraft cruise NO_*x*_ or lightning emissions), or chemistry cycling of oxidized nitrogen species to NO_*x*_. Overall, we highlight the effect of these possible sources on the differences between the modeled and observed NO_2_ VCD.

We use TROPOMI retrievals because of their higher resolution and availability of more temporal data than previous satellite instruments such as the Ozone Monitoring Instrument (OMI) and the Global Ozone Monitoring Experiment (GOME), coupled with their superior performance.^25^ Using simulated vertical profiles from high resolution models also helps improve satellite retrievals. CMAQ was selected as the CTM due to its heavy reliance by U.S. regulatory agencies such as the Environmental Protection Agency (EPA) in assessing emissions changes and the effectiveness of environmental policies, its capabilities to simulate species concentrations at higher grid resolutions, and its wide use in past studies. We also use direct measurements of EGU emissions^26^ which reduces the amount of uncertainty in CTM results. Of note, the term CTM when used herein refers specifically to CMAQ simulated results.

## 2 Methods

### 2.1 CMAQ Simulations of VCDs and NO_*x*_ Ratios

CTM results from Lawal et al.,^27^ which compared NO_2_ VCDs derived from the Community Multiscale Air Quality (CMAQv5.3.2) chemical transport model with TROPOMI VCD retrievals over Atlanta, Georgia, were used. A detailed description of CMAQ and its governing chemical and physical processes can be found in Byun and Schere.^15^ Modeled NO_2_ VCDs near two power plants (Bowen and Scherer) whose NO_*x*_ emissions are well characterized with CEMs^26^ were used to assess potential biases in TROPOMI. The WRF-SMOKE-CMAQ platform^15^ (Table S1) was used to simulate air quality over the 244 km × 244 km model domain (Figure 1), with a horizontal grid spacing of 4 km and 32 vertical layers extending to ∼16 km (100 hPa) during the month of August of 2019. Anthropogenic emissions were from the National Emissions Inventory 2016v1 Emissions Modeling Platform^28^ and include a modified airport emissions inventory which integrates NEI ground based airport emissions with full-flight (i.e., cruise) emissions from the Aviation Emissions Inventory Code (AEICv2.1) emissions repository.^29,30^ Lightning NO_*x*_ emissions were calculated using a statistical parametrization method.^31^ Additionally, simulations with several EGUs (Figure S1), conducted in different parts of the U.S. for a wintertime (January 2016) and summertime period (July 2016) were included for additional analysis.

**Figure 1:**
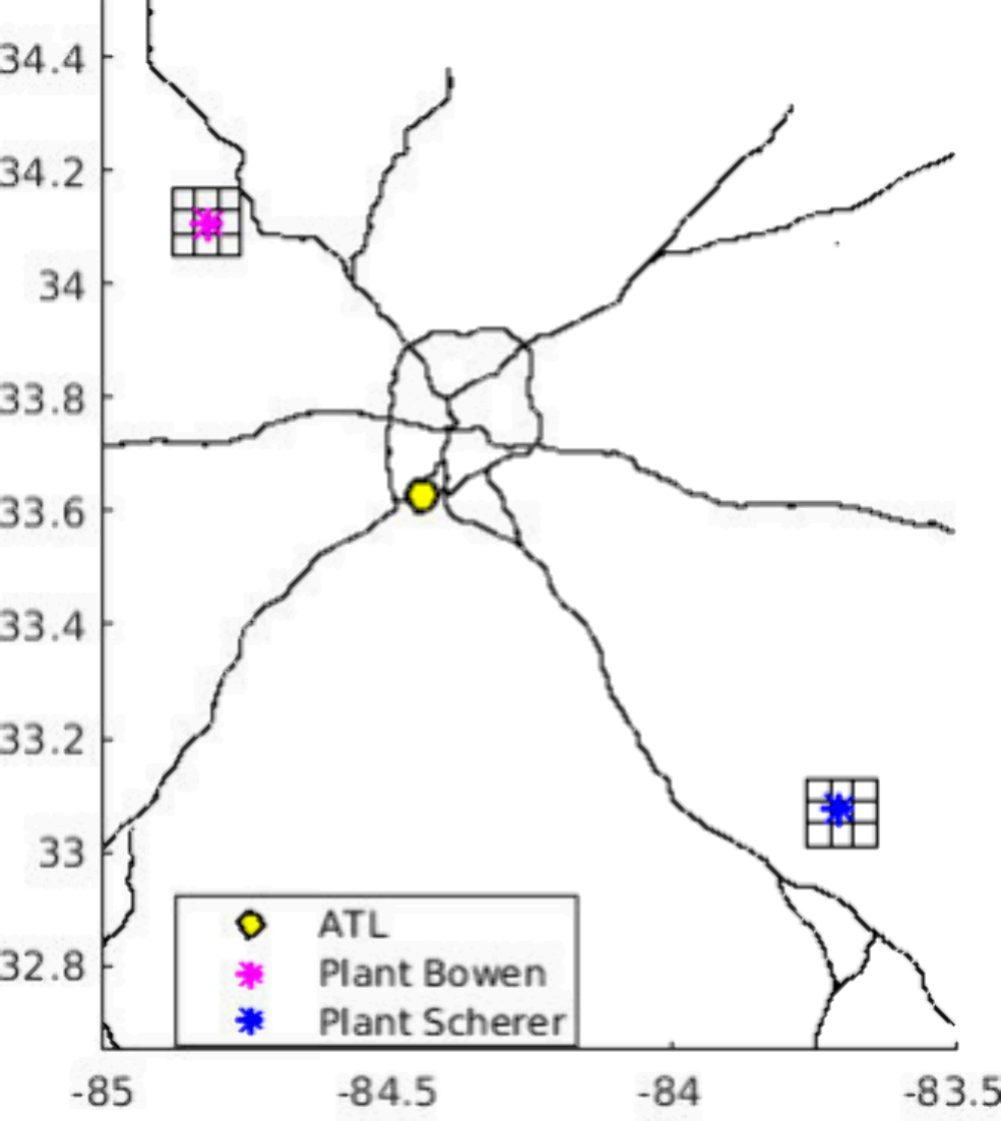
CMAQ modeling domain. Plant Bowen and Plant Scherer (both large electricity generation units) in northern Georgia (USA), with associated adjusted grids are shown, as are interstate highways. ATL is the Atlanta Hartsfield–Jackson International Airport.

### 2.2 TROPOMI NO_2_ Retrieval Process

TROPOMI follows a sun-synchronous orbit with an overpass time near 1:30 pm local solar time. The offline Level 2 v1.3 TROPOMI NO_2_ product developed by the Royal Netherlands Meteorological Institute (KNMI) accessed at NASA’s Goddard Earth Sciences Data and Information Services Center (GES DISC, https://tropomi.gesdisc.eosdis.nasa.gov/) was used in the study. Data are filtered to exclude pixels with solar zenith angles greater than 60°, quality flag (qa_value) less than 0.5, and cloud cover fraction (CLCF) criterion of less than 0.3, such that a total of 24 days out of the 31 days in August are used in this analysis. Seven of the 24 days had to be discarded due to missing data from pixels needed for analysis, resulting in a total of 17 days available for comparison of tropospheric TROPOMI and CMAQ NO_2_ VCDs. The level 2 (L2) product provides tropospheric VCDs, and the associated air mass factors (AMFs), which are calculated using vertical distributions from the TM5-MP model at 1° × 1°. In line with Cooper et al.,^32,33^ we used the provided averaging kernels and model vertical profiles from our higher resolution model to produce alternative AMFs which are then used to recalculate TROPOMI VCDs. For all CMAQ-TROPOMI tropospheric comparisons, TROPOMI L2 data were remapped to the CMAQ grid using oversampling.^27^

### 2.3 Gaussian Plume and Pseudosteady State Modeling (Gaussian-PSS)

The potential level of a CTM overestimating the conversion of NO to NO_2_ in an EGU plume was first evaluated by simulating plume growth using a Gaussian plume model of a NO_*x*_ containing plume into an atmosphere with pre-existing ozone levels, assuming a pseudosteady state balance (Gaussian-PSS) between O_3_, NO, and NO_2_. First, the NO_*x*_ concentrations in the plume are calculated using eq 1 where *x*, *y*, and *z* are the downwind, crosswind, and vertical locations relative to the effective emission location.

1 $$\left[NO_{x}\right]\left(x,y,z\right)=\frac{Q}{2\pi U\sigma_{y}\sigma_{z}}e^{-1/2{\left(\frac{y}{\sigma_{y}}\right)}^{2}}e^{-1/2{\left(\frac{z}{\sigma_{z}}\right)}^{2}}$$

*Q* is the source strength, *U* is the wind speed, and *σ*_*y*_ and *σ*_*z*_ are the horizontal and vertical diffusivities which are functions of *x*.^34^ Of note, the stability class and equations used to calculate *σ*_*y*_ and *σ*_*z*_, as referenced from Seinfeld and Pandis^35^ can be found in the accompanying Supporting Information (SI). This equation assumes that NO_*x*_ is conserved, recognizing that oxidation of NO_*x*_ to nitric acid and other photochemical species will occur relatively slowly in the plume. Of note, our analysis follows the plumes for about 2 h. Next, the NO_*x*_ concentrations are used in a pseudosteady state model (eqs 2–4), to calculate the distribution of NO, NO_2_, and O_3_ where [NO]_0_, [NO_2_]_0_, and [O_3_]_0_ are the initial levels of those species (i.e., levels that would occur without chemical conversion).

2 $$\left[O_{3}\right]\left(x,y,z\right)=-\frac{1}{2}\left({\left[NO\right]}_{0}\left(x,y,z\right)-{\left[O_{3}\right]}_{0}\left(x,y,z\right)+\frac{j_{NO2}}{k_{O_{3}+NO}}\right)+\frac{1}{2}{\left\{{\left({\left[NO\right]}_{0}\left(x,y,z\right)-{\left[O_{3}\right]}_{0}\left(x,y,z\right)+\frac{j_{NO2}}{k_{O_{3}+NO}}\right)}^{2}+4\frac{j_{NO2}}{k_{O_{3}+NO}}\left({\left[NO_{2}\right]}_{0}\left(x,y,z\right)+{\left[O_{3}\right]}_{0}\left(x,y,z\right)\right)\right\}}^{1/2}$$

3 $$\left[NO\right]\left(x,y,z\right)=\frac{j_{NO2}\left[NO_{x}\right]\left(x,y,z\right)}{k_{O_{3}+NO}\left[O_{3}\right]\left(x,y,z\right)+j_{NO2}}$$

4 $$\left[NO_{2}\right]\left(x,y,z\right)=\left[NO_{x}\right]\left(x,y,z\right)-\left[NO\right]\left(x,y,z\right)$$

Here *j*_NO__2_ is the photolysis rate of NO_2_, and *k*_O_3_+NO_ is the rate of reaction between NO and O_3_ to form NO_2_ and oxygen. In our case, we assume that the initial NO and NO_2_ concentrations are [NO]_0_ = 0.9*­[NO_*x*_]_0_ and [NO_2_]_0_ = 0.1*­[NO_*x*_]_0_, respectively, using measured ratios from a previous study^36^ and where [NO_*x*_] represents the total NO_*x*_ concentrations resulting from the EGU emissions. The resulting concentration distributions can be integrated numerically over the three-dimensional domain to find the fraction of the NO_*x*_ in the plume, that is NO.

As a test case, the NO:NO_2_ ratio in a plume was calculated as a function of distance using the above approach for an elevated plume using a slightly unstable atmosphere. We use the American Society of Mechanical Engineers (ASME) relationships^35^ between the dispersion coefficients as a function of *x*, a NO_*x*_ source strength of *Q* = 50 × 10^3^ kg/day (∼50 tons/day: a moderately sized power plant with emission controls), and a wind velocity of 5 m s^–1^ to obtain [NO_*x*_] from eq 1. To calculate the pseudosteady state ozone distribution (eq 2), we used a 1:30 pm approximate local time of the TROPOMI overpass in the southeastern US, leading to a *j*_NO__2_ value of 0.009 s^–1^ and an O_3_ + NO reaction rate constant (*k*_O_3_+NO_) of 2 × 10^–14^ cm^3^ mol^–1^ s^–1^. We set the initial ozone concentration [O_3_]_0_ to 60 ppb, similar to what might be expected on a summer day in the Southeast.^37^ The resulting concentration fields were then integrated over the plume from its origin downwind for horizontal distances in the *x* and *y* directions, between 1 and 10 km and up to 8 km in the vertical direction (*z*) to calculate the fraction of NO_*x*_ that is NO and NO_2_ (eq 3 and eq 4) in the plume. Of note, a similar analysis can be done for concentrated ground level sources, including highways (using a line-source model) and for larger areas with high levels of NO_*x*_ emissions (e.g., the urban cores of cities, airports, and rail yards). Such an analysis and application is more complicated as urban areas have a mix of source and source strengths and will be subject to different NO_*x*_ chemistry than might be observed in a plume from an EGU. Further, highways are not isolated, and downwind transport of NO_*x*_ from upstream sources is important. This is discussed in further detail in the SI.

Elshout et al.^36^ suggested that a combination of mixing and kinetics dynamics govern NO oxidation; however, Janssen et al.^19^ proposed that within a plume this process is largely kinetically driven. To this end, we use our Gaussian-PSS model to test these dynamics with three scenarios, one where the O_3_ levels are kept at 60 ppb and *U* is 5 m/s, a scenario where we lower ozone to 10 ppb, keeping *U* at 5 m/s, and the last, where O_3_ levels are kept at 60 ppb but wind speed is increased to 10 m/s. The results are in the Supporting Information and are discussed in the Results section.

Lastly, we demonstrate, using the Gaussian-PSS model, how chemical mischaracterization resulting from numerical dilution due to the Eulerian grid size of the CTM changes the distribution of modeled NO_*x*_ between NO_2_ and NO in the originating grid of a power plant plume.

### 2.4 Adjustment of NO_2_ in Upper-Tropospheric CTM Levels

Modeled rural NO_2_ from CTMs tends to be biased low when compared with aircraft and satellite-based observations, a trend that is consistent across studies despite differences in season, climate, and regions. These differences are also observed with other Eulerian based CTMs as well, such as GEOS-CHEM.^38^ These CTM biases could result from errors in atmospheric mixing, atmospheric reactions, and emission inventories. For example, potential errors related to tropospheric/stratospheric exchange, rapid chemical depletion in the model or an emissions bias (e.g., from aircraft or lightning),^39^ and other background sources such as chemical pathways converting oxidized nitrogen species to NO_*x*_ could account for the biases. Mischaracterization of NO_*x*_ from lightning which is particularly important for the month of August^40^ and aircraft emissions can account for a 30% to 40% increase in upper tropospheric NO_*x*_.^41^ Given the range of potential reasons why upper level NO_2_ levels may be biased low, adjustments of modeled simulated upper tropospheric NO_2_ (i.e., see Figures S2 and S3) are done as a sensitivity study here. Observations of upper tropospheric NO_2_—between 450 hPa and 180 hPa—using cloud slicing suggest that NO_2_ concentrations are usually between 50 and 100 ppt in the Southeastern United States during summertime (Marais et al.),^42^ though recent analyses suggest prior estimates of upper tropospheric NO_2_ might be higher (Shah et al.).^43^ In our baseline CTM simulation, we find that the average simulated NO_2_ concentrations in the upper troposphere are 30–40 ppt (Table S2). Thus, as a sensitivity study to determine the potential impact of errors from underestimated NO_2_ and missing emission sources, we recalculate the simulated upper tropospheric NO_2_ concentrations while keeping the simulated NO_*x*_ mass balance from the CMAQ base case constant at elevations above 8 km from ∼40 to ∼80 ppt. As a result, this adjustment falls more in line with Marais et al.’s^42^ observations of NO_2_ (i.e., ∼100 pt) within the uncertainty of the measurements.

### 2.5 Sensitivity Assessments for Modeled Domain

We evaluated how changes in the modeled NO:NO_2_ ratios of two power plant plumes and their adjacent grids (Figure 1), and upper tropospheric NO_*x*_ ratios at altitudes above ∼8 km, impacted the comparison with TROPOMI VCDs. Adjustments are first made to power plant plumes (up to ∼8 km) by using the estimated Gaussian plume calculations described in Section 2.3. NO_*x*_ ratio adjustments in the upper troposphere as described in Section 2.4 are then incorporated. New AMFs are calculated using the adjusted vertical NO_2_ profile studies.^32,44^ The effect of adjusted AMFs on NO_2_ VCDs between each CTM adjusted case and TROPOMI VCDs is evaluated using several statistical metrics such as the root-mean-square error (RMSE) and normalized mean bias (NMB). Temporal and spatial averaging of TROPOMI VCDs reduces the random component of the uncertainty associated with TROPOMI AMFs, and focus on difference metrics such as bias and RMSE minimizes the systematic component of error in this analysis.^45,46^

## 3 Results and Discussion

### 3.1 NO_*x*_ Ratio EGU Gaussian Plume Calculations vs CMAQ EGU NO_*x*_ Distributions

For the first 2 km of a plume (corresponding to a grid size of 4 km, assuming that, on average, the source is in the middle of the grid), approximately 90% of the NO_*x*_ will be in the form of NO (Figure S4). Results from the Gaussian–PSS model show that in each consecutive grid the fraction that is NO decreases as NO is oxidized to NO_2_ (Figure 2a). In contrast, in CMAQ, this conversion to NO_2_ happens rapidly within the same computational grid as that of the EGU plume source. The CMAQ-modeled NO_*x*_ ratio in the grid cell containing Plant Scherer (and similarly Plant Bowen) shows an ∼0.27/0.73 split between NO and NO_2_ (Figure 2b). Overall, it takes up to nearly 8 grid cells (∼30 km) from the source grid before the NO_*x*_ ratios in the Gaussian model approach those modeled by the CTM. When the vertical profiles of NO and NO_2_ ratios are plotted, relatively little vertical variation in the ratio is seen (Figure S5).

**Figure 2:**
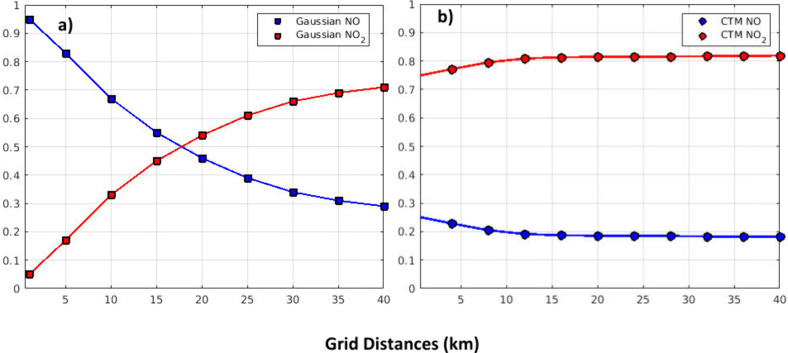
Fraction of the total NO_*x*_ that is NO and NO_2_ plotted at various distances from the plume source. a) NO and NO_2_ ratios calculated using the Gaussian plume and PSS approximation model. b) NO and NO_2_ ratios near Plant Scherer (Figure 1) as calculated from the CMAQ simulations.^27^

While there are limits to the plume dynamics calculations in the Gaussian–PSS model (e.g., the interaction with both the ground and top of the boundary layer, radial wind shifts), these results provide a more realistic representation of the actual NO_*x*_ ratios expected in a plume and are comparable to the findings in other similar modeling studies. In Melo et al.,^47^ for example, modeled results of exiting concentrations of a power plant plume found 49% of total NO_*x*_ to be NO_2_ at distances up to 40 km, matching measurements. Additionally, measurements of the fraction of NO_*x*_ as NO_2_ in plumes (up to ∼2.7 km) were no larger than 0.17. In Janssen et al.,^48^ measured NO:NO_2_ ratios were mostly below 0.5 at distances up to 10 km, only increasing at those distances if higher ozone mixing ratios and wind speeds (i.e., dilution) were observed. The results here can also be compared to other independent observations that show the core of a power plant plume at 11 km and 36 km downwind having greatly reduced ozone^37^ due to depletion through reaction with NO and in accord with the observations of NO:NO_2_ ratios in power plant plumes, lending further support to the calculated plume NO_*x*_ ratios presented here. Also, 2019 measurements of a plume from Plant Scherer found near equal levels of NO and NO_2_ within 20 km, supporting calculations in Figure 2a.^49^

Lastly, while the potential for the rapid numerical vertical dilution of NO_*x*_ to lead to high NO:NO_2_ ratios in CTM simulations is not fully quantified here, these results show that it readily explains much of the potential differences in the NO_2_ VCD comparisons. More detailed quantification could be addressed by using in situ measurements from airborne campaigns that acquire data within plumes, as well as high spatial resolution (i.e., 250 m) NO_2_ column density information from airborne spectrometers like the GEO-CAPE Airborne Simulator (GCAS).^50^ Further, conducting dispersion model calculations (e.g., using AERMOD^51^) for the urban area and comparing NO:NO_2_ ratios calculated using the Gaussian–PSS or more detailed large eddy simulations can be insightful.

We note that the artificially high rate of NO oxidation to NO_2_ within the plumes as a result of numerical dilution is not limited to those EGUs but can be seen for other EGUs within the CONUS modeling domain, located in different regions across the U.S. Figure S6 shows similarly modeled NO_*x*_ ratios for the Navajo Generating Station in Arizona and Plant Mountaineer, located in West Virginia. This is also observed during winter and summer months as well. A similar emission profile of NO and NO_2_ is shown with Plant Mountaineer and Plant Bowen in Figure S7.

### 3.2 Plume Dynamics in CMAQ and Gaussian–PSS Models

The physical and chemical processes in plumes such as those originating from power plants differ from ambient (regional) air chemistry and processes, and concentrations of NO, NO_2_ and O_3_, and OH will be different from the magnitudes discussed in Valin et al.,^18^ where the focus was on urban plumes. As noted in Elshout et al.,^36^ NO oxidation of power plant plumes is strongly dependent upon mixing, and under ambient conditions, high oxidation rates of NO due to ozone entrainment near the plume edges are likely to be observed. In the center of the plume, it is more likely that the level of the O_3_ will be low due to depletion from high NO concentrations, so less oxidation will occur. We model the relationship between turbulent mixing and oxidant ratios with the Gaussian–PSS model by comparing the three scenarios discussed in the methods in Section 2.3. Results presented in Figure S8 show that when O_3_ is set at 60 ppb NO oxidations to NO_2_ under two wind speeds (Figure S9­(a), *U* = 5 m/s, and Figure S7­(c), *U* = 10 m/s) are similar. When the levels of O_3_ are reduced to 10 ppb (Figure S8­(b)), NO oxidation is considerably reduced, and a large proportion of NO_*x*_ remains largely as NO near the source. This has important consequences when using satellite observations to help identify and quantify the emissions from large NO_*x*_ sources, including power plants and airports because on days with low ozone only a small fraction of the NO will be converted to NO_2_, and the model bias in the NO_2_ to NO_*x*_ ratio can be higher on low ozone days. Similarly, on days when the atmosphere is more stable, the plume would remain more concentrated near the source, leading to less of a NO-to-NO_2_ transformation.

### 3.3 Impact of Numerical Dilution and Diffusion

The Gaussian–PSS model described in Section 2.3 is used to model NO_*x*_ within the originating grid of a power plant plume to illustrate how chemical mischaracterization resulting from numerical dilution due to the Eulerian grid size of the CTM changes the distribution of modeled NO_*x*_ between NO_2_ and NO. The results of this case study with the Gaussian plume model are presented in Figure 3. Changes in NO_*x*_ distribution for different CTM grid dimensions of *x* and *y* (i.e., Figure 3a) are plotted for three different emission rates of NO_*x*_. Initially, NO/NO_2_ ratios are similar, irrespective of the emission rates at smaller grid sizes (Figure 3b), but then the results show an increasing conversion of NO to NO_2_ as grid size changes. In all cases, an increase in NO_*x*_ conversion to NO_2_ is seen as grid size increases irrespective of emissions flux in the concentrated NO_*x*_ plume. Further detailed discussion about the model applications can be found in the SI.

**Figure 3:**
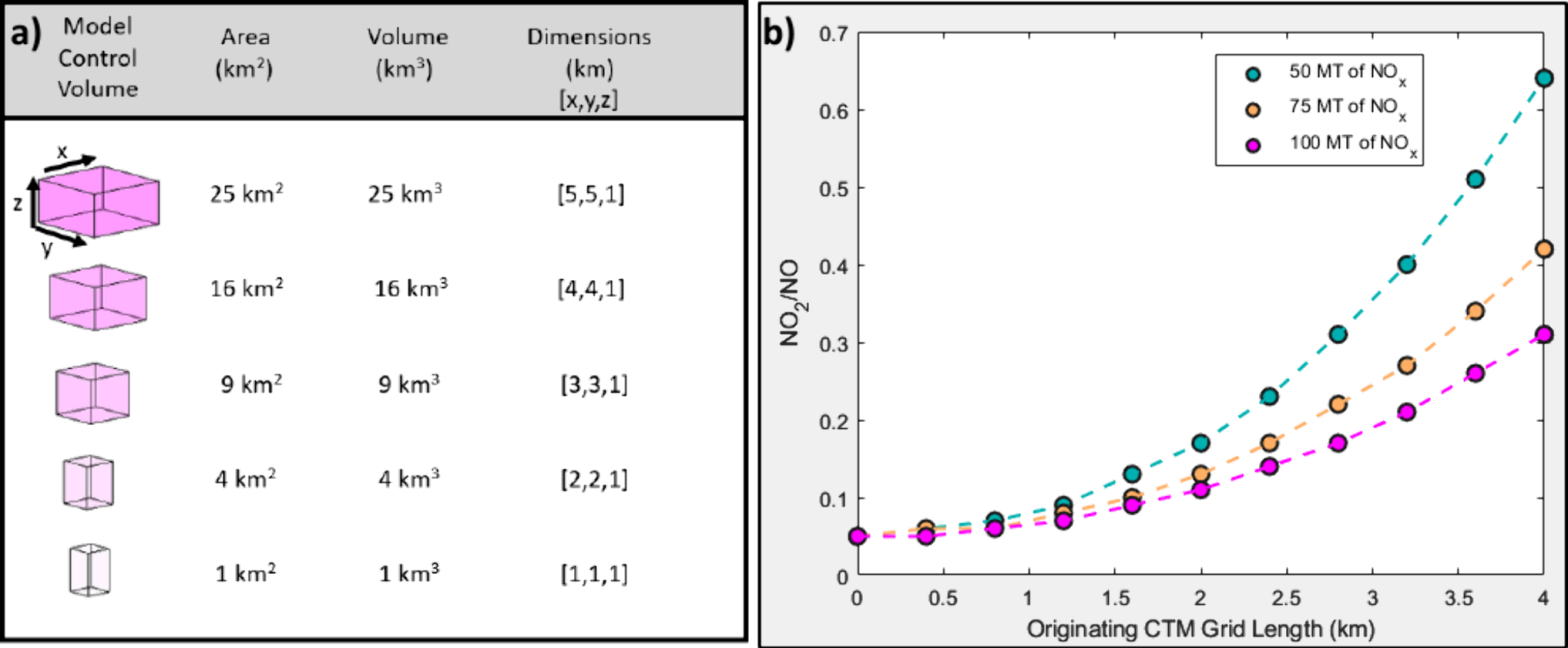
a) Changes in model grid volume as dimensions in *x* and *y* are adjusted. b) Shows for three different emission rates how NO_2_ increases steadily as the grid sizes are adjusted to what is shown in (a).

### 3.4 NO:NO_2_ Ratio Adjustments on Simulated CMAQ NO_2_ VCDs of Power Plants

In the recent comparison of TROPOMI-derived NO_2_ VCDs and CMAQ simulations conducted using a 4 km grid resolution in the Atlanta, Georgia, USA, area, the TROPOMI-derived NO_2_ VCDs were about 50% of those derived using CMAQ (Figure 4a).^27^ In Goldberg et al.,^52^ a similar disagreement between TROPOMI and a CTM^53^ was found near the Martin Lake, TX, power plant, though they posit that modeling parameters (i.e., NO_2_ chemical and dispersion lifetime) in combination with model resolution are a potential source of the discrepancy. Here, we attempt to correct for the rapid numerical dilution effect on NO to NO_2_ oxidation as if the numerical control volume was representative of the actual process, by imposing the 2/3 NO/NO_*x*_ ratio as observed near the source in Janssen et al.^19^ in the boundary layer in the vertical column directly above the power plant and 1/2 in the vertical columns of the eight grids adjacent to the grid with the power plant. This is done with the understanding that emissions from EGU plumes primarily dominate NO_*x*_ within the boundary layer of the column directly above the EGU. Based on reaction kinetics and experimental measurements, total NO_*x*_ would largely exist as NO near the source. Further, gaseous oxidants such as O_3_ are rapidly depleted in such a plume, due to high NO concentration, thus limiting further conversion of NO to NO_2_. The eight adjacent grids were selected because for the most part the simulated plumes around each power plant appeared to persist only for those adjacent grids, dissipating to a steady profile beyond the 8 km radius around each plant with most experimental models showing at best a steady NO_2_/NO_*x*_ ratio of 0.5 to 0.6 downwind of the source.^19,36^ A similar idea involves averaging NO_*x*_ around point sources horizontally rather than vertically to preserve the strong NO_*x*_ gradients observed and constrain the NO_*x*_ ratios.^54^ In this case, it was found, after recalculation of the AMFs, that the RMSE at both sites is decreased on average by 30% (Figure 4b and Table S3; Bowen: 2.0 to 1.4 × 10^15^ molecules/cm^2^; Scherer: 1.8 to 1.2 × 10^15^ molecules/cm^2^).

**Figure 4:**
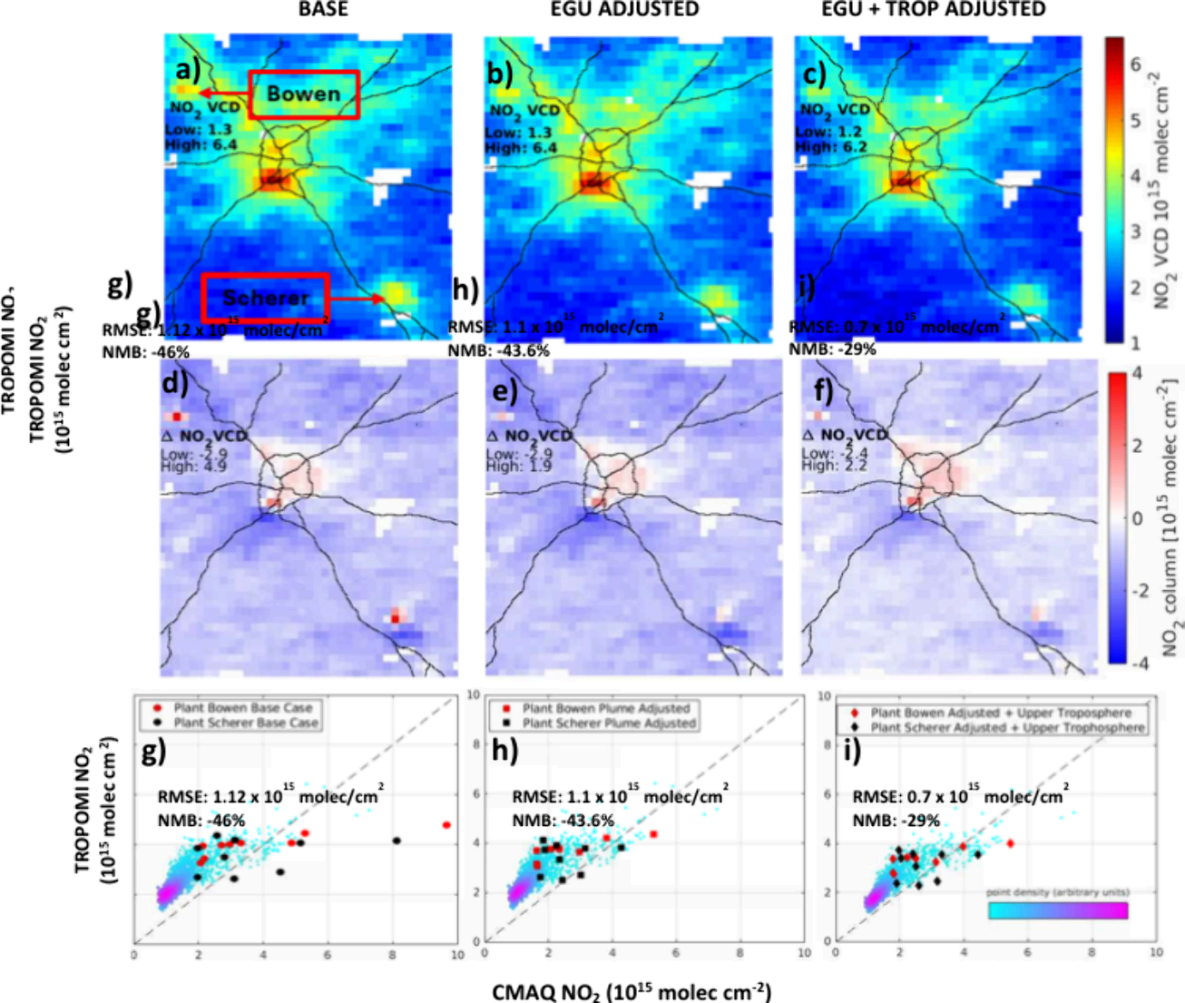
a–c) TROPOMI NO_2_ VCD plots. d–f) CMAQ-TROPOMI NO_2_ VCD difference plots. g–i) Respective density scatter plots from scenarios in figures d to f. (a,d,g) Represent the base case with no adjustments for potential NO:NO_2_ model differences in NO_*x*_ elevated plumes at two EGUs (Plant Bowen and Plant Scherer) and in the upper troposphere. (b,e,h) Show adjustments of NO_*x*_ bias in elevated plumes at the two EGUs. (c,f,i) Show adjustments of NO_*x*_ bias in elevated plumes at both EGUs and at higher altitudes above 8 km. Black lines are Interstate highways. The red and black dots in (g–i) represent the EGUs and the surrounding 8 grid cells as depicted in Figure 1. Data from the plots are averaged over the 17 selected days in August 2019.^27^

There is further evidence that artificial dilution could be a large source of the discrepancies between CTM calculated NO_2_ VCDs and TROPOMI (or other satellite-based products) near large NO_*x*_ sources when looking at spatial biases between the two. For instance, the degree of difference between the two diminishes downwind from the source fairly quickly for both the power plants and over the city (Figure 4d and 4e). If the NO_*x*_ mass flux was constantly biased high or low in the model simulations, a persistent bias downwind of the sources would be found as most of the NO would be converted to NO_2_. Looking specifically at the two power plants from the initial base case, the highest differences were found just over the sources and rapidly dissipated downwind. Further, at one of the largest and more dispersed NO_*x*_ sources in the model region, the Atlanta airport (Figure 1), there is a large positive difference right over the airport itself, but that also goes away within one or two surrounding adjacent grid cells. Over such small distances (i.e., 2, 3, or 4 km grid cells), a little of the NO_*x*_ would have been converted to nitric acid, suggesting not only that the modeled mass emissions of NO_*x*_ are approximately correct and conserved but also that at distances further away where most of the NO_*x*_ is NO_2_ (i.e., observed negative bias) even lower modeled NO_2_ is observed. This lends further credence to the hypothesis that CTMs overestimate the NO to NO_2_ conversion very near the source, an effect of artificial dilution. The model also has lower than observed NO:NO_2_ ratios when compared to ground observations in high source regions, also suggesting more rapid NO to NO_2_ oxidation (Figures S9 and S10).

### 3.5 Impact of Upper Troposphere NO_2_ Adjustments

Differences in modeled and TROPOMI VCDs extend beyond areas near highly emitting NO_*x*_ sources. Modeled NO_2_ VCDs tend to be lower than those of satellite-based observations (Figure 4). This could be partly due to an underestimate in background NO_2_, which could include an underestimate of stratospheric NO_*y*_ transport or an underestimate of biogenic/geogenic NO_*x*_ emissions, both which have an increasing contribution to total NO_*x*_ as anthropogenic NO_*x*_ emissions are reduced.^55^ A follow up study^56^ indicated that inventory inputs to CTMs were likely missing a significant portion of nonanthropogenic background emissions that could contribute to more NO_2_ partitioning. For example, additional (i.e., beyond that included in the current modeling) lightning-induced NO_*x*_ could account for some unaccounted for NO_2_.^39,57^ Regional CTM emission inventories that do not include cruise emissions could also lead to underestimates of NO_2_ in the upper troposphere as well.^27,58^ Both can help explain why modeled NO_2_ VCDs in more rural areas are lower than the observations.

Here, in addition to the EGU plume adjustments, we increased the average upper tropospheric NO_2_ above 8 km from ∼30 ppt (Figure S2) to ∼80 ppt (Figure S3) to be more in line with Marais et al.^59^ This level of adjustment is also in line with Shah et al.’s^43^ findings of 1 × 10^14^ molecules/cm^2^ uncertainty with model simulations. When this modification is made, the RMSE is reduced from 1.12 to 0.70 × 10^15^ molecules/cm^2^. In Figure 4i, the overall NMB is reduced from −43% to −29% (Table 1). The grids where a high bias in CMAQ remains are near the Atlanta airport and downwind of the urban core.

**Table 1:** Tabulated Performance Metrics of NO_2_ Vertical Column Densities of CMAQ and TROPOMI^a^

Adjustments	Data cut off	NMB %	Absolute bias 10^15^ molecules/cm^2^	Mean CMAQ 10^15^ molecules/cm^2^	Mean TROPOMI 10^15^ molecules/cm^2^	Slope	Pearson correlation	RMSE 10^15^ molecules/cm^2^	No. of data points
CMAQ_Initial_	All Data	–43	–1.04	1.4	2.5	0.76	0.81	1.12	2665
	<4.5	–44	–1.05	1.4	2.5	0.88	0.82	1.25	2644
	≥4.5	22	0.93	5.5	4.6	0.17	0.29	2.63	21
EGU plume adjustments	All Data	–43.6	–1.0	1.4	2.5	0.82	0.82	1.1	2665
	<4.5	–43.9	–1.1	1.4	2.5	0.87	0.82	1.2	2649
	≥4.5	10.4	0.45	5.2	4.8	0.53	0.57	0.70	16
Upper Tropospheric adjustments (>8 km)	All Data	–28.5	–0.61	1.6	2.2	0.79	0.85	0.72	2665
	<4.5	–29.0	–0.62	1.6	2.2	0.92	0.86	0.50	2639
	≥4.5	28.2	1.12	5.5	4.3	0.16	0.27	2.92	26
EGU and Upper Tropospheric (>8 km) adjustments	All Data	–29	–0.61	1.6	2.2	0.85	0.86	0.70	2665
	<4.5	–29	–0.62	1.6	2.2	0.91	0.86	0.49	2644
	≥4.5	17	0.70	5.3	4.5	0.54	0.57	0.98	21

a Results were tabulated using the represented number of grid points (Figure 1) for the 17 selected simulation days and are separated into three categories: all NO_2_ VCD data points, NO_2_ VCDs less than 4.5 × 10^15^ molecules/cm^2^, and NO_2_ VCDs greater than 4.5 × 10^15^ molecules/cm^2^.

### 3.6 AMF Differences

Lastly, we look at the changes in AMF from the adjustments made in this study. Here we compare the results from the base case with the combined EGU NO_*x*_ ratio adjustment and the upper-tropospheric NO_2_ adjustments. Distributions of AMFs from the base (initial) case and the recalculated AMFs from the adjustments (Figures S11 and S12, respectively) show that the adjustment led to a slight increase in average AMFs (i.e., 0.88 to 1.0). This 15% increase in the AMF, albeit small, led to a substantial decrease in overall NMB (Table 1) and a greater spread over the 1–1 line (Figure 4i). The effect of this change was even more notable than the differences in CMAQ results between the base CMAQ case (i.e., 3D Base Case) and a default inventory (Table S4).^27^

### 3.7 Limitations

This study is limited in its analysis to one satellite instrument and one computational Eulerian model. While variability in results might occur from different mixing regimes, inputs and chemical mechanisms among different models, the numerical dilution effect is common to Eulerian models when subgrid scale emissions are simulated^19,20,60^ and this artifact is likely to be observed in other Eulerian models without a Plume-in-Grid treatment.^61,62^ Another concern is the short time span used in this analysis (i.e., August 2019) and whether the results would be impacted if a longer time frame was used. As this study directly builds upon the findings of the previous study,^27^ it was necessary to use the same time frame in our assessment. It is also worth noting that the numerical effect of artificial dilution in CTMs is not affected by the choice of the season, region, or time span (see discussion in the SI). Simulations of multiple EGUs conducted at different times (i.e., 2016 vs 2019, summer vs winter) support the generality of the findings. Future work should consider the incorporation of a Plume-in-Grid model in an Eulerian model and evaluation of these effects at distances that extend beyond plume origins.^24^ With the availability of future satellite products at higher resolution than TROPOMI, such as TEMPO,^50^ additional analysis done at higher resolutions of CTMs will be insightful.

Lastly, while this paper focuses mainly on modeled NO_2_, it is likely that there will also be some impact on OH and O_3_, both of which react with NO_*x*_, although the extent is not specifically addressed or quantified here. However, a change in the NO_2_ could lead to changes in the modeled O_3_ in the power plant plume. Another effect, primarily near the source, is that modeled NO_2_ will be more widely dispersed, leading to higher O_3_ and OH levels, increasing HNO_3_ formation and aerosol nitrate.

### 3.8 Implications

The comparison of modeled and satellite observed NO_2_ VCDs highlights the importance of accounting for differences in modeled NO:NO_2_ ratios that stem from artificial plume dilution near large stack emissions. For instance, the change in normalized mean bias (NMB) as shown in Table 1 (NMB: 22% to 10.4%) suggests that the seemingly large difference between TROPOMI and CTM VCDs over high emitting NO_*x*_ sources could be partially due to overly rapid NO to NO_2_ conversion in the model. Not accounting for the effect of artificial dilution can result in modeled NO_2_ VCD^63^ bias, where increases in the NO_2_:NO_*x*_ from 0.5 to as high as 0.8 were seen under highly convective conditions. Missing NO_*x*_ emissions could degrade the comparison as well.^56^

One approach to adjust for differences occurring from numerical diffusion in CTMs is to use a Plume-in-Grid model within the CTM to account for rapid dispersion effects over the computational grid;^62^ however, there are still limitations with those solutions, one being the computational requirements for finer-scaled applications.^61^ It is also important to consider similar treatment for other ground-based high-NO_*x*_ emitting sources besides EGUs. For instance, NO_*x*_ emissions from sources such as airports, where landing and takeoff emissions are spatially categorized as ground level emissions in regulatory models, should more realistically be treated as elevated-plume sources as well.^28,64–66^ Further, to address potential errors in modeled upper troposphere mixing ratios, given the uncertainty in the NO:NO_2_ estimates, postsimulation recomputation of CTM-calculated VCDs can be employed as was done here. However, using observed measurements when available may introduce uncertainty. Of further note, to address the potential bias in the CTM-modeled NO_2_ VCDs over nonpoint sources with high levels of surface emissions, further analysis is recommended (e.g., using a model with a detailed ground-level dispersion approach or using observations if available).

While this paper focuses mainly on how rapid artificial model dilution impacts the NO:NO_2_ ratio and the resulting comparison between modeled and observed VCDs, the rapid dilution would also impact local OH, O_3_, and nitrate levels. The relative impact of mischaracterized physical processes such as convective transport and missing emission sources was also assessed in this study. Findings show that emission sources and oxidant chemistry could reduce model biases in the upper troposphere and rural areas.

This analysis shows that unless the columnar NO:NO_2_ ratio is captured by the model using fine-scale model calculations to compare with VCD observations, developing a top-down emissions inventory could result in erroneous estimates for strong NO_*x*_ sources. These results also provide further support that CTM simulations of NO_2_ in the upper troposphere are biased low. Further analysis of how uncertainties in the modeled NO_2_ impact using satellite NO_2_ observations for emissions inventory assessment and top-down inventory development is an important area for future research.

## Supplementary Material



## Notes

Additional details on methods, equations, materials; a map of EGU NO_*x*_ emissions, including plotted vertical profiles of simulated NO_*x*_ ratios from CMAQ, tabulated simulated NOx, tabulated results of TROPOMI, and CMAQ NO_2_ vertical column densities; and additional figures showing CMAQ and TROPOMI VCDs and recalculated AMFs (PDF)

## b

U.S. Environmental Protection Agency, Research Triangle Park, NC 27709, USA
