# Supplementary material for: Potential Errors in CMAQ NO:NO2 Ratios and Upper Tropospheric NO2 Impacting the Interpretation of TROPOMI Retrievals
Source: ACS EST Air. 2025 Apr 29;2(6):998–1008. doi: 10.1021/acsestair.4c00198 (PMC12172011; doi:10.1021/acsestair.4c00198)
Supplement: Supplementary file 1 [file ea4c00198_si_001.pdf]

Supplemental Information

Potential Errors in CMAQ NO:NO_2 Ratios and Upper Tropospheric NO_2 impacting the Interpretation of TROPOMI retrievals.

Abiola S. Lawal[1, 2,a], T. Nash Skipper[1,b], Cesunica E. Ivey[2], Daniel L. Goldberg[3], Jennifer Kaiser[1, 4], and Armistead G. Russell[1*]

[1]School of Civil & Environmental Engineering, Georgia Institute of Technology, 790 Atlantic Drive, Atlanta, GA 30332-0355, United States.

[2]Department of Civil and Environmental Engineering, 760 Davis Hall, University of California, Berkeley, CA 94720-1710, United States.

[3]Department of Environmental and Occupational Health, George Washington University, 950 New Hampshire Ave, Washington, DC, 20052, United States.

[4]School of Earth and Atmospheric Sciences, Georgia Institute of Technology, 790 Atlantic Drive, Atlanta, GA 30332-0355, United States.

.

Figures: 13

Tables: 4

Figures

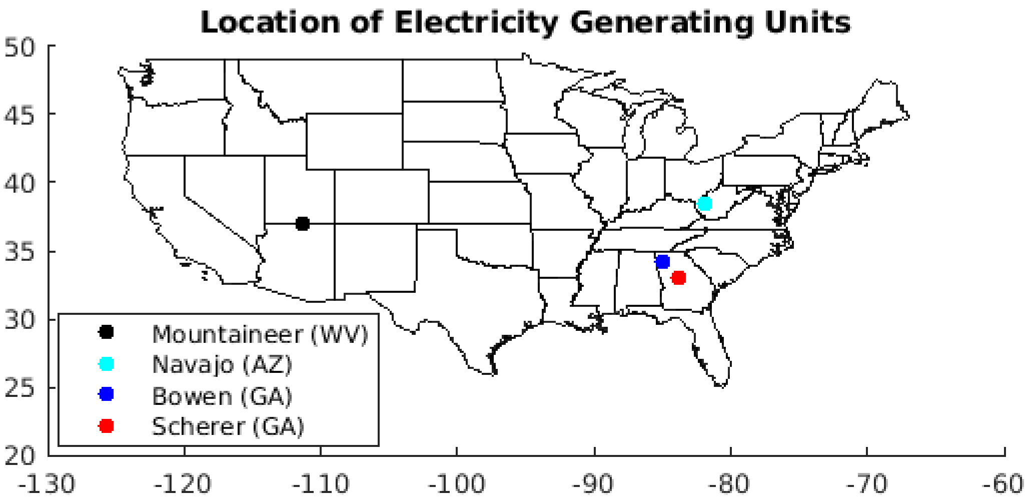

Figure S1. Location of electricity generating power plants (EGUs) domain considered in this study.

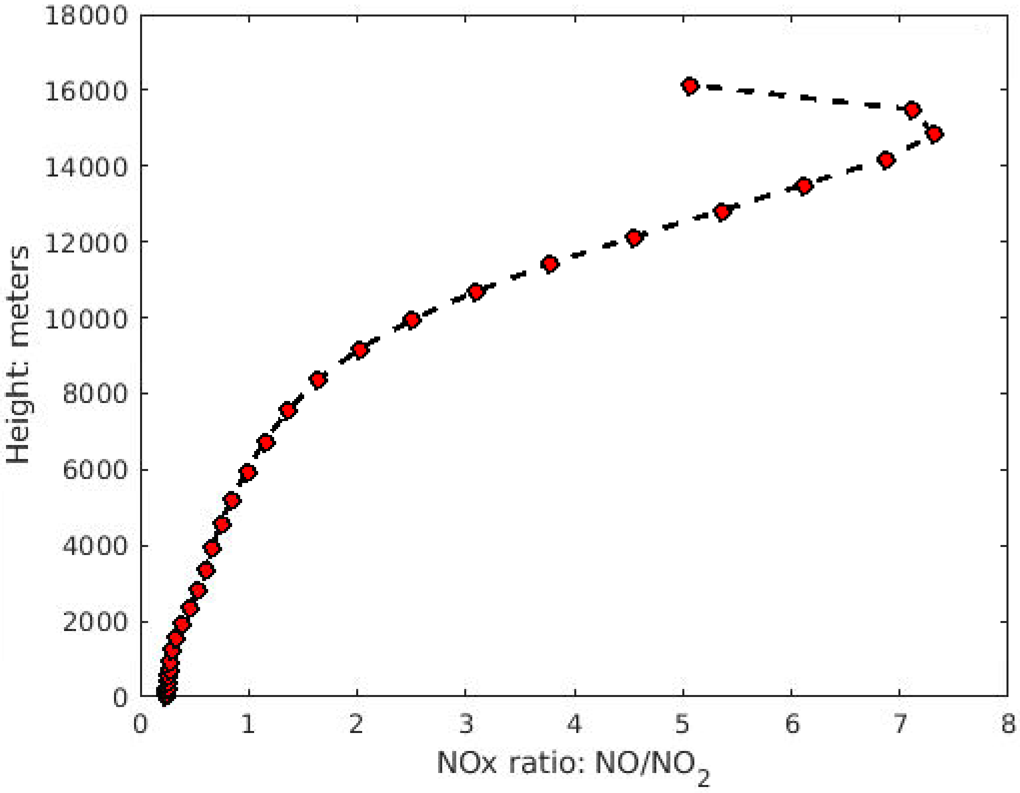

Figure S2. Averaged monthly NO:NO_2 simulated NO_x ratio as detailed in Lawal et al., (2022)[1].

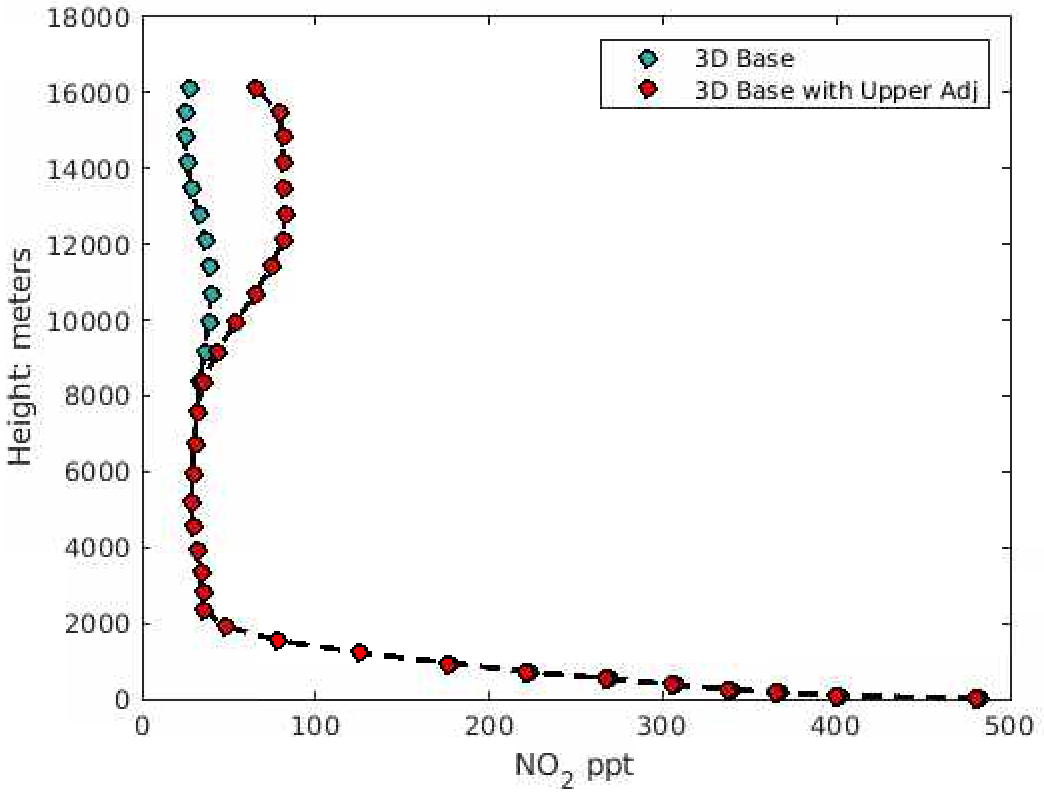

Figure S3. Adjustments to NO_2 proportions above 8km.

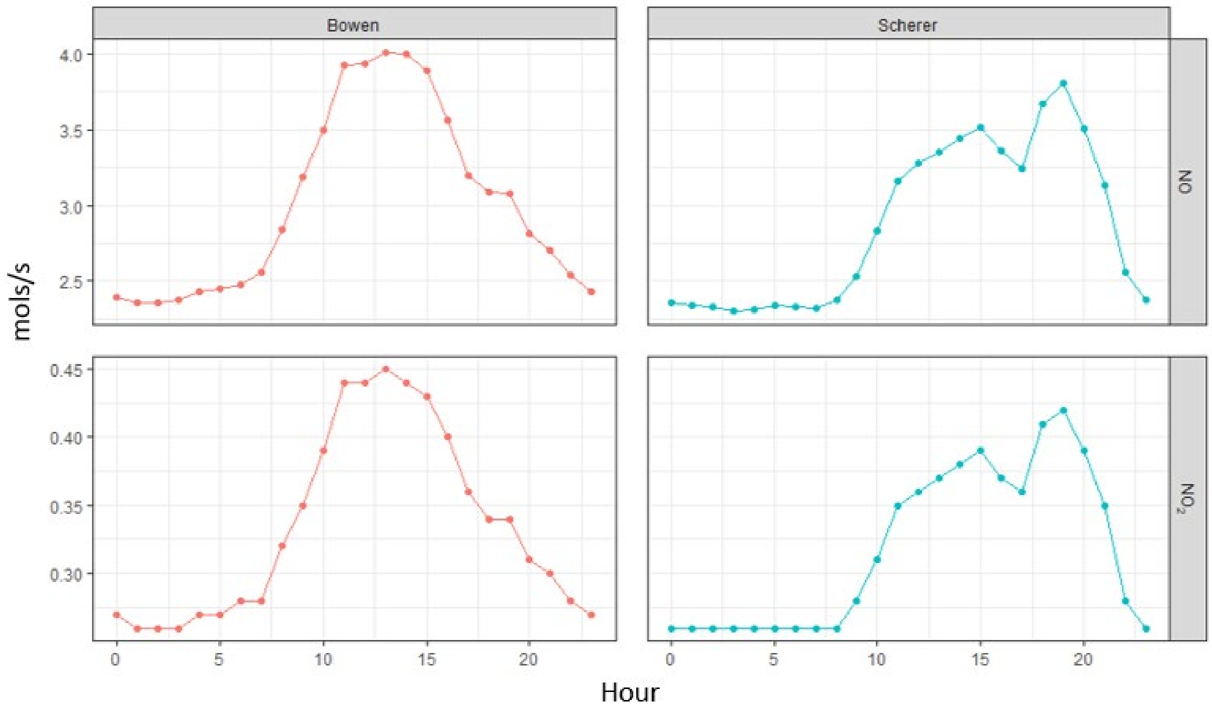

Figure S4. Average hourly NO and NO_2 emissions from Plant Bowen and Plant Scherer for the month of August 2019.

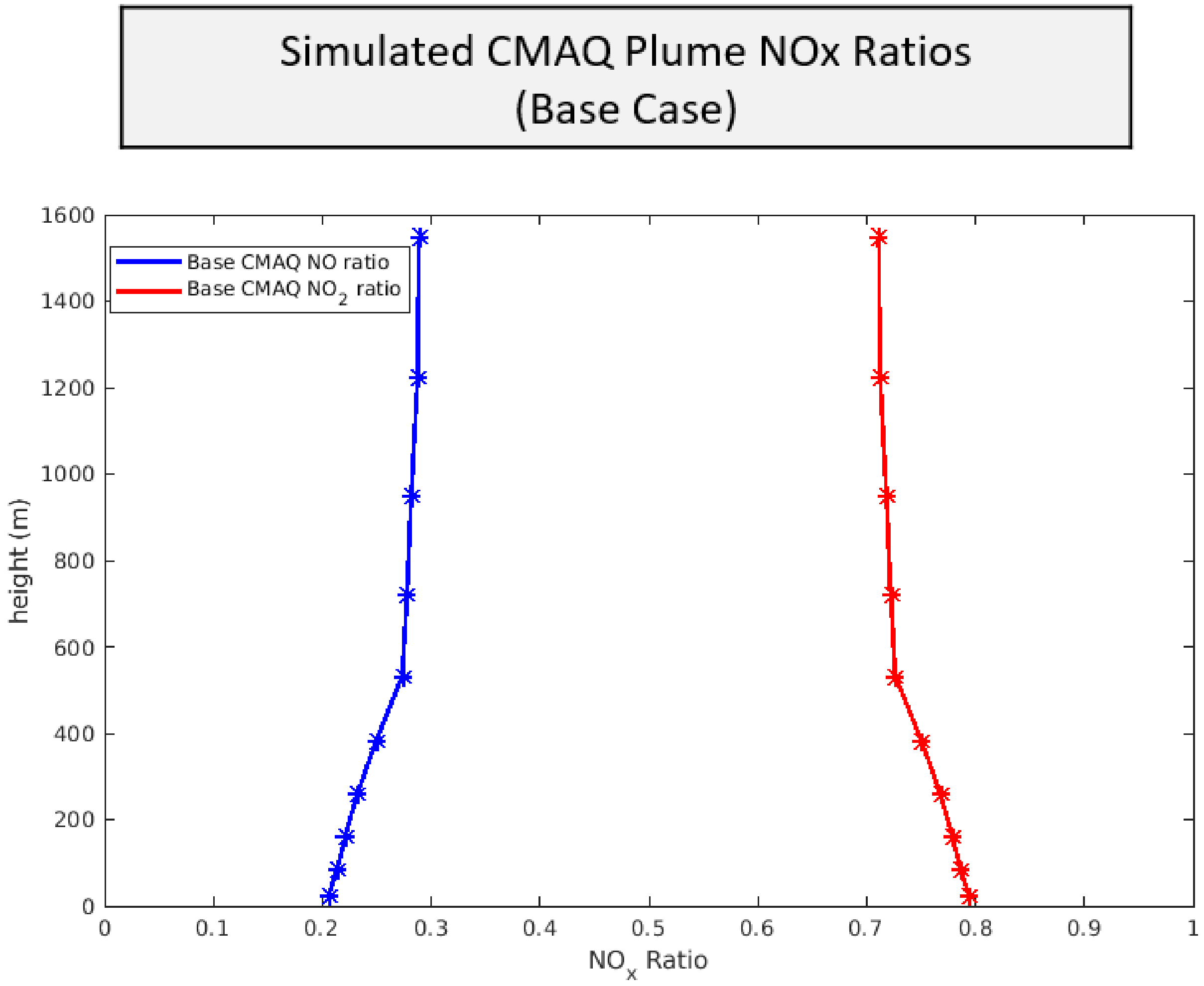

Figure S5: Modeled vertical profile of NO:NO_2 ratios at the originating grid for Plant Bowen. Taken from CMAQ simulated modeled mixing ratios of NO and NO_2 from Lawal et al., (2022)[1].

EGU Plant	January	July
West Virginia<br>**Mountaineer**	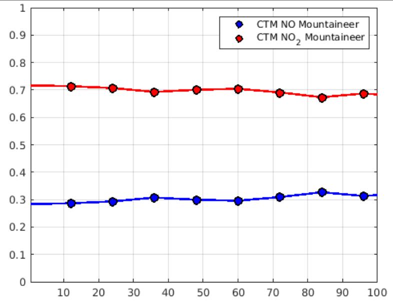	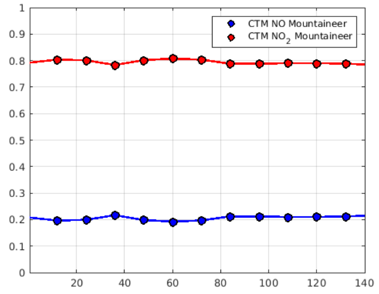
Georgia<br>**Plant Bowen**	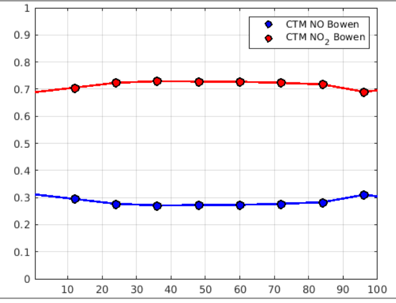	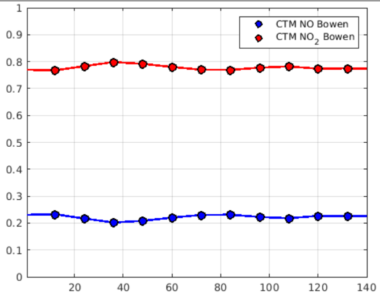
Arizona<br>**Navajo Generating Station**	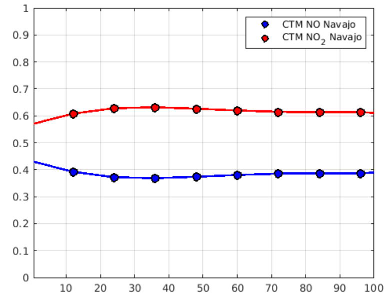	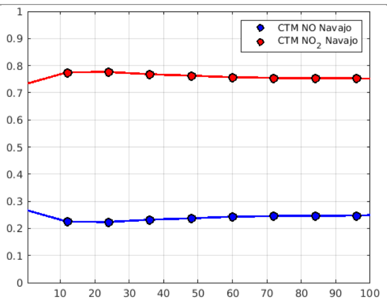

Figure S6. Fraction of the total NO_x that is NO and NO_2 plotted at various distances from the plume source as calculated from CMAQ simulations conducted during 2016 for January and July.

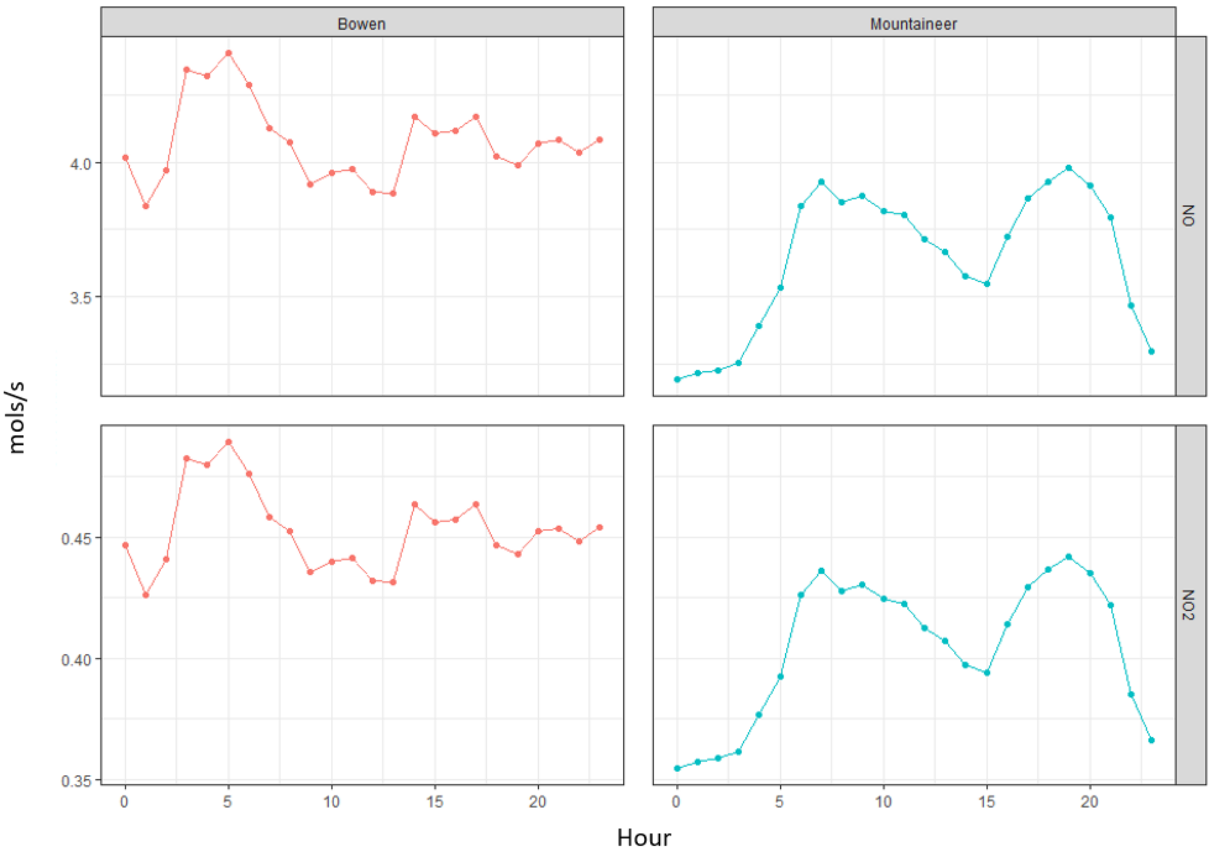

Figure S7. Average hourly NO and NO_2 emissions from Plant Bowen and Plant Mountaineer for the month of July 2016 obtained from CMAQ EGU emissions.

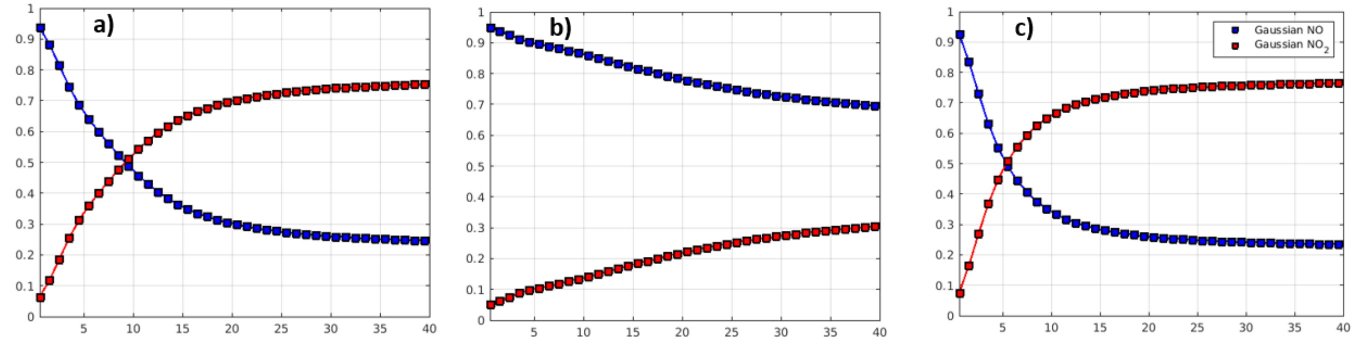

Figure S8 Fraction of the total NO_x that is NO and NO_2 plotted at various distances from the plume source as modeled from Gaussian-PSS Model.

a) O_3 = 60 ppb, U = 5 m/s, b) O_3 = 10 ppb, U = 5 m/s c), O_3 = 60 ppb, U = 10 m/s

Denoted O_3 concerntrations reflect initial O_3 concerntrations at the start of the model run.

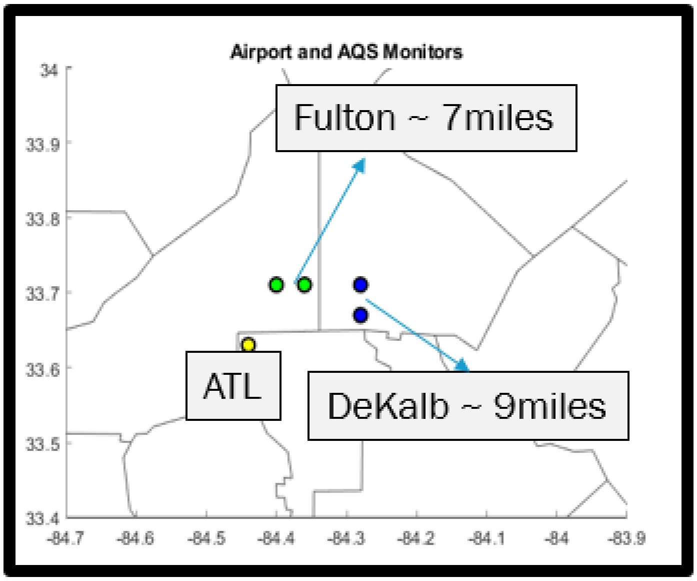

Figure S9. Location of air quality monitors at Fulton and DeKalb counties and their proximity to the Atlanta Hartsfield Jackson airport (ATL) in the modeling domain.

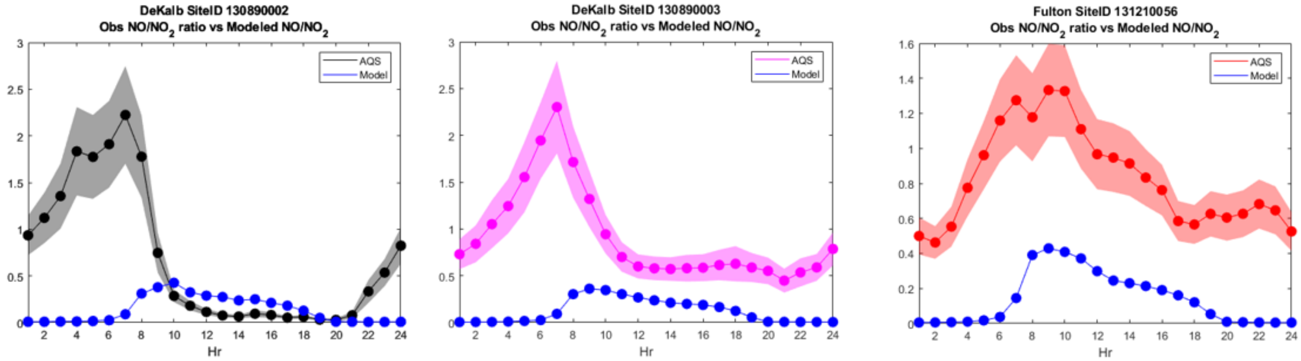

Figure S10. NO:NO_2 ratios at the three monitors shown in Figure S9. Shaded areas represent uncertainty.

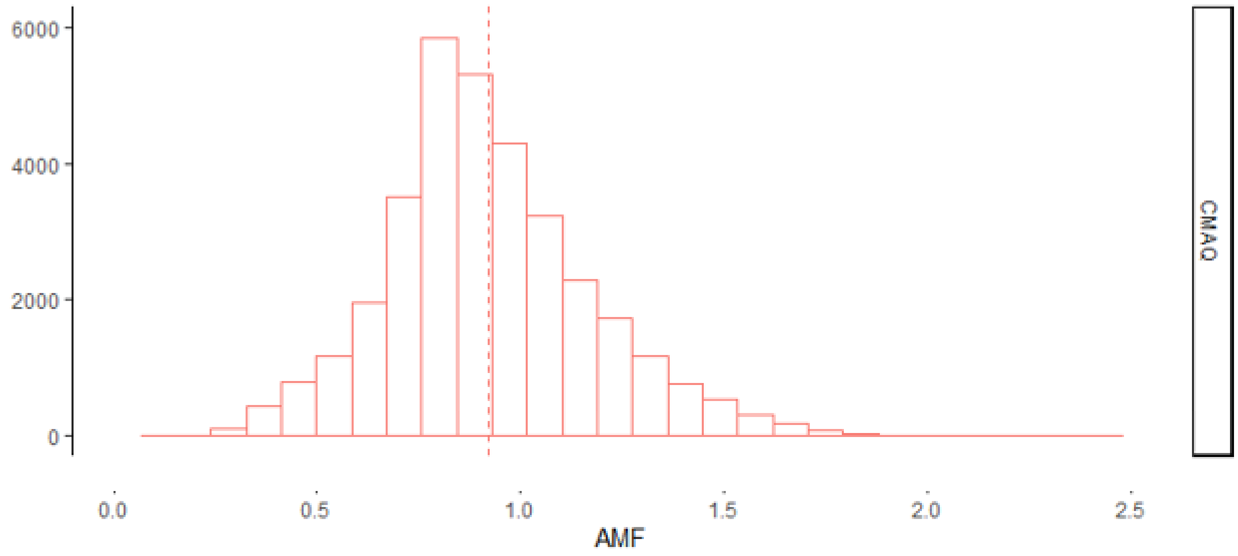

Figure S11. Histogram of AMFs as calculated from the initial base case in Lawal et al., (2022)[1].

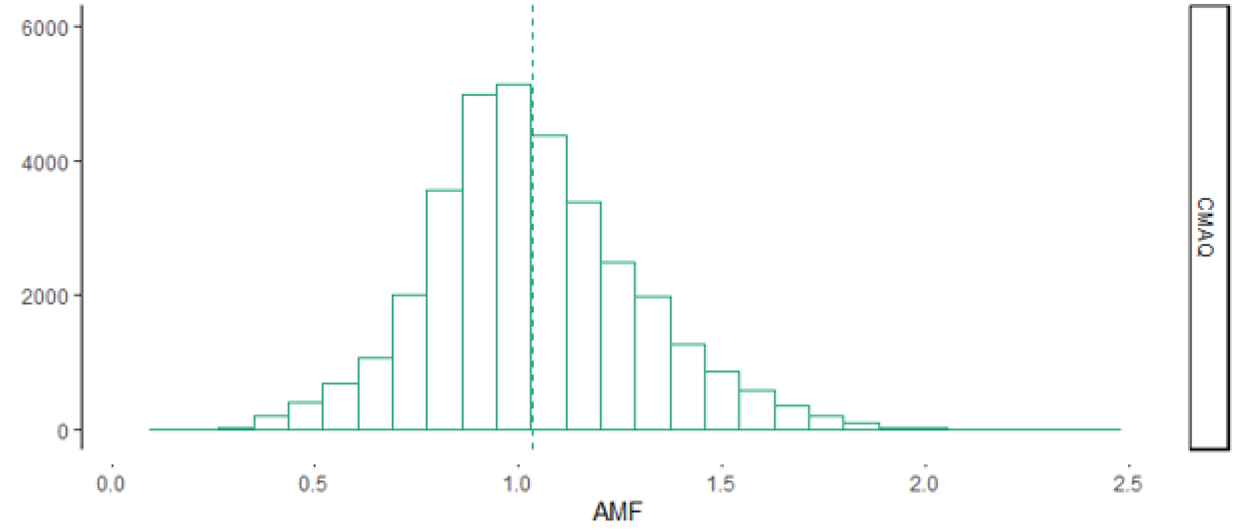

Figure S12. Histogram of AMFs as calculated after making adjustment to initial base case in Lawal et al., (2022)[1]. Changes here include adjustments to $NO:NO_2$ ratios above 8km. Ratios are adjusted to match the findings in Marais et al., (2021)[2] and adjustment of NO_x bias in elevated plumes at the two EGUs to match the Gaussian plume calculations as presented in this study.

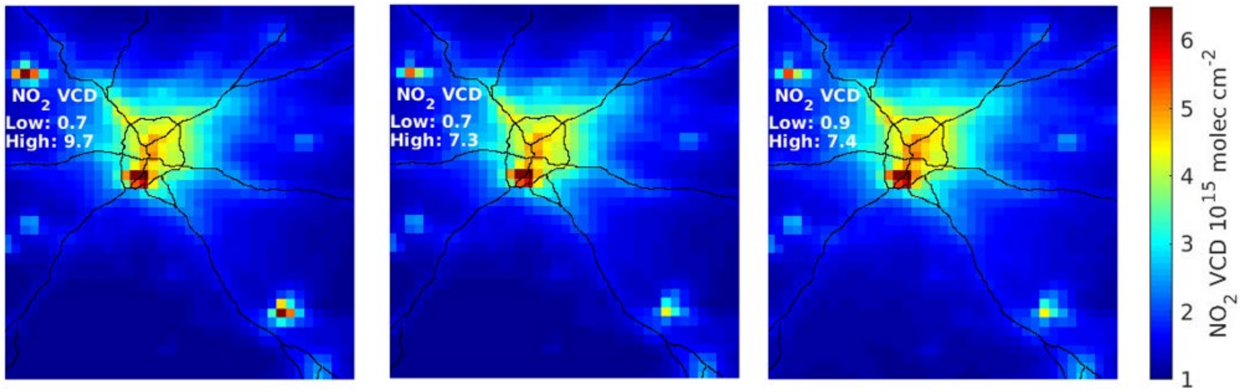

Figure S13. CMAQ NO_2 VCD plots averaged over the 17 selected days in August 2019. a) 3D base case from Lawal et al., (2022) b) adjustment of NO_x bias in elevated plumes at the two EGUs c) adjustment of NO_x bias in elevated plumes at both EGUs and at higher altitudes above 8km

Tables

Table S1. WRF-SMOKE-CMAQ Configurations

CMAQ	
CMAQ version	**V5.3.2**
Chemical Mechanism	**cb6r3_ae7_aq**

Emission Platform	
NEI Emissions Platform	**2016**
SMOKE Platform	**V4.7**

WRF	
WRF version	**V4.1.2**
Land Surface Scheme	**Noah Land-Surface Model**
Longwave Radiation	**CAM**
Shortwave Radiation	**CAM**
Microphysics	**Morrison 2-moment**
Cumulus parameterization	**Kain-Fritsch**
PBL Scheme	**YSU**

Table S2. Tabulated concentrations of NO, NO_2 from the Lawal et al., 2022[1] obtained from the modeling domain at each vertical height. (i.e. 32 vertical levels up to 100 hpa). Also included is the total NO_x and NO:NO_2 ratio. Results are tabulated and averaged over the month of August 2019[1].

NO (ppb)	NO_2(ppb)	NO_x (ppb)	NO:NO_2 ratio	Height (meters)	Case Name
0.11	0.48	0.59	0.22	26	3D Base
0.09	0.40	0.49	0.22	86	3D Base
0.08	0.37	0.45	0.23	163	3D Base
0.08	0.34	0.42	0.24	260	3D Base
0.08	0.31	0.38	0.25	381	3D Base
0.07	0.27	0.34	0.25	533	3D Base
0.06	0.22	0.28	0.26	721	3D Base
0.05	0.18	0.23	0.27	950	3D Base
0.04	0.13	0.16	0.29	1224	3D Base
0.03	0.08	0.10	0.33	1549	3D Base
0.02	0.05	0.07	0.39	1926	3D Base
0.02	0.04	0.05	0.46	2356	3D Base
0.02	0.03	0.05	0.53	2835	3D Base
0.02	0.03	0.05	0.60	3361	3D Base
0.02	0.03	0.05	0.66	3932	3D Base
0.02	0.03	0.05	0.74	4551	3D Base
0.02	0.03	0.05	0.85	5222	3D Base
0.03	0.03	0.06	0.98	5948	3D Base
0.03	0.03	0.06	1.14	6731	3D Base
0.04	0.03	0.07	1.36	7549	3D Base
0.05	0.03	0.09	1.64	8361	3D Base
0.07	0.04	0.11	2.01	9153	3D Base
0.10	0.04	0.13	2.49	9925	3D Base
0.12	0.04	0.16	3.08	10678	3D Base
0.15	0.04	0.19	3.77	11412	3D Base
0.17	0.04	0.20	4.55	12126	3D Base
0.17	0.03	0.21	5.36	12824	3D Base
0.17	0.03	0.20	6.11	13505	3D Base
0.18	0.03	0.20	6.87	14174	3D Base
0.18	0.02	0.20	7.32	14833	3D Base
0.17	0.02	0.20	7.10	15489	3D Base
0.14	0.03	0.16	5.06	16145	3D Base

Table S3. Tabulated quantitative metrics of NO_2 vertical column densities for Bowen and Scherer. Results were tabulated using the represented number of grid points (Figure 1) for the 17 selected simulation days.

Adjustments	EGU	NMB %	Absolute Difference 10^{15} molecules/ cm^2	Mean CMAQ 10^{15} molecules/ cm^2	Mean TROPOMI 10^{15} molecules/ cm^2	Slope	Pearson Correlation	RMSE 10^{15} molecules / cm^2	No of Data Points
**$CMAQ_{initial}$**	**Bowen**	-6.0	-0.09	3.9	4.0	0.16	0.84	1.97	9
**EGU plume**		-31.3	-1.10	2.6	3.7	0.27	0.82	1.4	9
**Upper Tropospheric Adjustments (> 8 km)**		7.0	0.42	4.1	3.7	0.16	0.84	2.0	9
**EGU + Upper Tropospheric Adjustments (> 8 km)**		-19.2	-0.58	2.8	3.4	0.27	0.81	1.05	9
**$CMAQ_{initial}$**	**Scherer**	4.1	0.12	3.71	3.58	0.10	0.29	1.78	9
**EGU plume**		-22.9	-0.84	2.5	3.4	0.09	0.11	1.2	9
**Upper Troposphere Adjustments (> 8 km)**		16.8	0.54	3.9	3.3	0.12	0.35	1.8	9
**EGU + Upper Troposphere (> 8 km)**		-10.9	-0.4	2.7	3.1	0.12	0.17	0.95	9

Table S4. Tabulated quantitative metrics of NO_2 vertical column densities for both inventories across the domain. Results were tabulated for the 17 selected simulation days. Results here are from Lawal et al., (2022)[1].

Inventory	Data cut off	NMB %	Absolute Difference 10^{15} molecules/ cm^2	Mean CMAQ 10^{15} molecules/ cm^2	Mean TROPOMI 10^{15} molecules/ cm^2	Slope	Pearson Correlation	RMSE 10^{15} molecules / cm^2	No of Data Points
**Default**	All Data	-46	-1.13	1.4	2.5	0.74	0.79	1.21	2665
	< 4.5	-47	-1.15	1.3	2.5	0.92	0.82	1.45	2644
	>= 4.5	29	1.36	6.1	4.7	0.19	0.48	4.09	21
**3D Base Case**	All Data	-43	-1.04	1.4	2.5	0.76	0.81	1.12	2665
	< 4.5	-44	-1.05	1.4	2.5	0.88	0.82	1.25	2644
	>= 4.5	22	0.93	5.5	4.6	0.17	0.29	2.63	21

Discussions

Section 1

The similar issue of computational dilution and rapid dispersion can be found, and analysis conducted, when considering concentrated ground level sources, e.g., highly trafficked areas, including sources in ports (shipping, rail, airports) and highways, though such sources are not so concentrated. Similar to how the Gaussian plume equation is derived in Section 2.3, if the turbulent atmospheric diffusion follows a linear profile near the group (see Seinfeld and Pandis (1998), chapter 18)[3], the steady-state atmospheric diffusion equation for a line source can be solved as

$$[NO_x](x,z) = \frac{Qb}{Ux} e^{-\left(\frac{Uz}{bx}\right)}$$

Where, the vertical diffusivity is given as:

$$K_{zz} = bz$$

x is the downwind distance, z the vertical distance, *b* a diffusivity parameter, U the velocity (assumed constant) and Q the source strength (g m^{-1} s^{-1}). Calculating the ozone, NO and NO_2 concentrations from this solution are done the same as in section 2.3 (equations 2 to 4).

Given an isolated freeway, with a source strength of Q, and a diffusivity parameter b. Plotting the ratio of average NO to NO_x as a function of plume distance from length from the freeway will show a similar distribution to Figure 2a. While power plants are often more isolated, the case with freeways in a city is much more complicated as there are multiple roads and other sources of NO_x that interact. This would lead to even more of the NO_x being found as NO_2.

NO is typically smaller as found by comparing NO/NO_2 ratios near freeways vs. those further away. For example, NO:NO_2 ratios observed at two near-road monitors (DeKalb) in Atlanta were compared to those at a site approximately 1 km from a freeway in a forested area (Fulton) at 13:00 LST (the approximate time of the flyover) as seen in Figure S10.

The ratios were 0.24 and 0.24 at the two near road monitors and 0.24 at the more distant site (Figure S9). Given an ozone level of about 40 ppb (~1x10^8), a 1:00 pm approximate time of the TROPOMI fly-over in the southeastern US), J_{NO2} value of 0.009 s^{-1} and an O_3+NO recombination rate of 2x10^{-14} cm^3 mol^{-1} s^{-1}, the ratio of NO_2 to NO should be about 4.5, so most of the NO_x in the model will be NO_2 if the plume is well mixed with the regional ozone.

Section 2

Numerical Diffusion Impacts

A general negative model bias persists in NO_2 satellite retrievals (i.e. TROPOMI and OMI), when compared to NO_2 profiles modeled by Eulerian Chemical Transport models in highly polluted NO_x regimes[4, 5]. Previous studies that explore the sources of these biases, have largely focused on satellite retrievals themselves or the retrieval algorithms, as opposed to those emanating from CTMs as the main source of these biases. For instance, differences in cloud parameters such as cloud height, fraction and mask used in retrieval algorithms, as noted in Wang et al., (2020)[6] are a possible source of bias differences among different satellite products and model comparisons. Different algorithms have also been tested to improve retrievals as evidenced from studies that have considered the impact of algorithm changes[7]. Other studies consider discrepancies that could occur from using different surface albedo data sets which could affect the estimation of AMFs[8-10]. It is important to note that while many of these studies have shown improvement through exploration of atmospheric properties and retrieval algorithms, the low biases in TROPOMI when compared with CTMs continues to persist and is not yet wholly understood.

This study explores how misrepresentation of the CTM NO_2 shape profiles from NO_x kinetic mischaracterization of CTM due to numerical dilution can affect the satellite retrievals. This approach is different from most studies that focus largely on atmospheric properties (i.e. cloud fraction etc.) that affect satellite retrievals. We select a specific case, region, and season to highlight a potential source of error and bias when deriving and comparing satellite retrievals with modeled vertical column densities of NO_2 from grid-based (Eulerian Models) Chemical Transport Models (CTMs). The selection is based on the previously study by Lawal et al., (2022)[1], that used a select number of days from the month of August, 2019. Although a potential limitation, the choice to utilize a select number of days in this study does not affect the conclusions drawn here for several reasons. One of the main reasons is that the premise of our study is based on the well documented negative bias in NO_2 satellite retrievals (i.e. TROPOMI and OMI), when compared to NO_2 profiles modeled by Chemical Transport Models like CMAQ in highly polluted NO_x regimes. This global bias is consistent, despite differences in season and climate amongst different regions in these studies that use CTM derived NO_2 VCD in comparison with satellite retrievals and does not appear to be affected by length of model days.

The dilution effects of grid-based models on modeled concentrations are well documented and common to grid-based Eulerian models, and is therefore, expected to remain constant, irrespective of the domain, season, or temporal range. This is because the chosen size of the model grid can affect the magnitude of emission flux (and thereby it's concentration) mathematically beyond what is actually taking place in the process. Due to setup of CMAQ as a grid based Eulerian model, all the sources within the grid are assumed to be well mixed over the control volume of the model grid, thus mathematically diluting concentrations of NO_x sources homogenously within the grid instantaneously. The resulting adjustment in the emission flux due to this numerical/mathematical treatment is an unintended change in chemical kinetics and the modeled output. In essence, like in our case for example where the grid length is 4 km, NO_x which is emitted from a point source (EGU), with an area footprint of approximately 1 km^2, is treated in an Eulerian grid-based model as being uniformly distributed over an area of 16 km^2 (16:1 ratio) as its originating collocated grid, changing the true chemical concentration available in that grid and thus the chemical kinetics. Thus, a high NO_x plume that would not normally be widely dispersed in said manner, will now be numerically diluted over a larger and within the entire volume of the model grid. In essence, a point source is now treated as an area source in the CTM. This dilution effect holds through all regions and seasons and is not expected to change over any range of days. Our findings and results here also match those found in the following studies of Valin et al., (2011)[11] and Gillani et al., (1996)[12], which are referred to in the main manuscript.

We demonstrate, using the Gaussian-PSS model described in section 2.3, to model NO_x in the originating grid of a power plant plume to illustrate how chemical mischaracterization resulting from numerical dilution due to the Eulerian grid size of the CTM changes the distribution of modeled NO_x between NO_2 and NO. While the Gaussian-PSS approach presents a much-simplified treatment of NO_x

chemistry as it cannot capture all the complexity represented in CMAQ, it is an effective tool to explore the impact of Eulerian computational dilution on NO_x chemistry for several reasons. One of the reasons is the ease at which we are able to test the impact of different grid sizes, hence different control volumes, hence numerical dilution on NO_x kinetics. Another reason why the model does not necessarily need the complexity in CMAQ is because it is used mainly on a plume and in a single grid where the plume, a highly concentrated NO_x source is located. Therefore, we are not necessarily simulating complex atmospheric chemistry. Thus, while other the key factors in addition to NO_x emissions, like temperature, velocity and radical concentrations, all of which can be adjusted in the equation represented in the Gaussian-PSS, are not necessarily tested. Lastly, the theories of diffusion and concentration which are explored with the Gaussian-PSS model are not only well documented chemistry, but are also validated by measurements of NO_x plumes near the source and at distances further away[13]. The latter is an important point because the behavior of NO_x chemistry in the power plant plume as noted in Elshout et al., (1984)[13] is similar to what is modeled in the Gaussian Plume model, demonstrating its applicability to model power plants of different capacities, under various conditions.

The results of this case study with the Gaussian plume model are presented in section 3.3 (Figure 3) of the main manuscript.

Equations

Evaluation metrics with CMAQ base cases

$$NMD = 100\% \times \frac{\sum_i^n (3D_i - 2D_i)}{\sum_i^n 2D_i}$$

$$RMSE = \sqrt{\frac{\sum_{i=1}^N (3D_i - 2D_i)^2}{N}}$$

Note: $3D_i$ (CMAQ predicted values with 3D inventory), $2D_i$ (CMAQ predicted values with default inventory)

Evaluation metrics with CMAQ and observations

$$NMB = 100\% \times \frac{\sum_i^n (P_i - O_i)}{\sum_i^n O_i}$$

$$RMSE = \sqrt{\frac{\sum_{i=1}^N (P_i - O_i)^2}{N}}$$

$$NRMSE = \frac{\sqrt{\frac{\sum_{i=1}^N (P_i - O_i)^2}{N}}}{\overline{O_i}}$$

Note: P_i (CMAQ predicted values), O_i (AQS or TROPOMI)

NMD: Normalized Mean Difference

RMSE: Root Mean Square Error

NMB: Normalized Mean Bias

NRMSE: Normalized Root Mean Square Error

Gaussian Plume Equation Parameters

$$\sigma_y(ix) = 0.4 \times \{x(ix)\}^{0.91}$$

$$\sigma_z(ix) = 0.4 \times \{x(ix)\}^{0.91}$$

Where:

σ_y and σ_z are the standard deviations in meters, of wind velocity fluctuations in the y and z directions

(ix) is the representative model grid in CMAQ's modeling domain with a range from of 1 to 61.

$\{x(ix)\}$ is the distance in meters calculated as a function of the model grid.

Equations are as adapted from ASME (1973) from Seinfeld and Pandis, Table 18.3, stability category A

REFERENCES

(1) Lawal, A. S.; Russell, A. G.; Kaiser, J. Assessment of Airport-Related Emissions and Their Impact on Air Quality in Atlanta, GA, Using CMAQ and TROPOMI. *Environmental Science & Technology* **2022**, *56* (1), 98-108. DOI: 10.1021/acs.est.1c03388.

(2) Marais, E. A.; Roberts, J. F.; Ryan, R. G.; Eskes, H.; Boersma, K. F.; Choi, S.; Joiner, J.; Abuhassan, N.; Redondas, A.; Grutter, M.; et al. New observations of NO2 in the upper troposphere from TROPOMI. *Atmos. Meas. Tech.* **2021**, *14* (3), 2389-2408. DOI: 10.5194/amt-14-2389-2021.

(3) Steinfeld, J. I. Atmospheric Chemistry and Physics: From Air Pollution to Climate Change. *Environment: Science and Policy for Sustainable Development* **1998**, *40* (7), 26-26. DOI: 10.1080/00139157.1999.10544295.

(4) Kim, H. C.; Kim, S.; Lee, S. H.; Kim, B. U.; Lee, P. Fine-Scale Columnar and Surface NOx Concentrations over South Korea: Comparison of Surface Monitors, TROPOMI, CMAQ and CAPSS Inventory. *Atmosphere* **2020**, *11* (1). DOI: ARTN 101

10.3390/atmos11010101.

(5) Allen, D. J.; Pickering, K. E.; Pinder, R. W.; Henderson, B. H.; Appel, K. W.; Prados, A. Impact of lightning-NO on eastern United States photochemistry during the summer of 2006 as determined using the CMAQ model. *Atmos. Chem. Phys.* **2012**, *12* (4), 1737-1758. DOI: 10.5194/acp-12-1737-2012.

(6) Wang, C. J.; Wang, T.; Wang, P. C.; Rakitin, V. Comparison and Validation of TROPOMI and OMI NO(2)Observations over China. *Atmosphere* **2020**, *11* (6). DOI: ARTN 636

10.3390/atmos11060636.

(7) van Geffen, J.; Eskes, H.; Compernolle, S.; Pinardi, G.; Verhoelst, T.; Lambert, J. C.; Sneep, M.; ter Linden, M.; Ludewig, A.; Boersma, K. F.; et al. Sentinel-5P TROPOMI NO2 retrieval: impact of version v2.2 improvements and comparisons with OMI and ground-based data. *Atmospheric Measurement Techniques* **2022**, *15* (7), 2037-2060. DOI: 10.5194/amt-15-2037-2022.

(8) De Smedt, I.; Pinardi, G.; Vigouroux, C.; Compernolle, S.; Bais, A.; Benavent, N.; Boersma, F.; Chan, K. L.; Donner, S.; Eichmann, K. U.; et al. Comparative assessment of TROPOMI and OMI formaldehyde observations and validation against MAX-DOAS network column measurements. *Atmos Chem Phys* **2021**, *21* (16), 12561-12593. DOI: 10.5194/acp-21-12561-2021.

(9) Bauwens, M.; Compernolle, S.; Stavrakou, T.; Muller, J. F.; van Gent, J.; Eskes, H.; Levelt, P. F.; van der A, R.; Veefkind, J. P.; Vlietinck, J.; et al. Impact of Coronavirus Outbreak on NO(2)Pollution Assessed Using TROPOMI and OMI Observations. *Geophysical Research Letters* **2020**, *47* (11). DOI: ARTN e2020GL087978

10.1029/2020GL087978.

(10) Li, M.; McDonald, B. C.; McKeen, S. A.; Eskes, H.; Levelt, P.; Francoeur, C.; Harkins, C.; He, J.; Barth, M.; Henze, D. K.; et al. Assessment of Updated Fuel-Based Emissions Inventories Over the Contiguous United States Using TROPOMI NO2 Retrievals. *Journal of Geophysical Research-Atmospheres* **2021**, *126* (24). DOI: ARTN e2021JD035484

10.1029/2021JD035484.

(11) Valin, L. C.; Russell, A. R.; Hudman, R. C.; Cohen, R. C. Effects of model resolution on the interpretation of satellite NO2 observations. *Atmos Chem Phys* **2011**, *11* (22), 11647-11655. DOI: 10.5194/acp-11-11647-2011.

(12) Gillani, N. V.; Pleim, J. E. Sub-grid-scale features of anthropogenic emissions of NOx and VOC in the context of regional Eulerian models. *Atmospheric Environment* **1996**, *30* (12), 2043-2059. DOI: Doi 10.1016/1352-2310(95)00201-4.

(13) Elshout, A. J.; Beilke, S. Oxidation of No to No2 in Flue Gas Plumes of Power Stations. In *Physico-Chemical Behaviour of Atmospheric Pollutants*, Dordrecht, 1984//, 1984; Versino, B., Angeletti, G., Eds.; Springer Netherlands: pp 535-543.
